# Supplementary material for: Modeling critical thermoelectric transports driven by band broadening and phonon softening
Source: Nat Commun. 2024 Jan 26;15:776. doi: 10.1038/s41467-024-45093-6 (PMC10817957; doi:10.1038/s41467-024-45093-6)
Supplement: Supplementary file 1 — Supplementary Information [file 41467_2024_45093_MOESM1_ESM.pdf]

Supplementary Information

Modeling Critical Thermoelectric Transports Driven by Band Broadening and Phonon Softening

Kunpeng Zhao[1,2,#,], Zhongmou Yue[1,3,#], Hexige Wuliji[2], Hongyi Chen[4,*], Tingting Deng[1,3], Jingdan Lei[2], Pengfei Qiu[1,3], Lidong Chen[1,3], Xun Shi[1,3,*]*

A: Basic of the Landaus theory on order-disorder phase transitions[1, 2]

According to the Landaus theory, the order-disorder phase transition could be described by the order parameter ξ. In our case, the low temperature α-phase in Cu_2Se is a low symmetry ordered phase, and the order parameter has a nonzero value at the beginning of the phase transition. The high temperature β-phase is a high symmetry disordered phase with $\xi = 0$. During the phase transition, the order parameter changes continuously from nonzero to zero. Considering the neighborhood of the critical temperature T_p, the thermodynamic potential $\Phi(T, \xi)$ could be expanded in powers of ξ

$$\Phi(T,\xi) = \Phi_0(T) + A_1\xi + A_2\xi^2 + A_3\xi^3 + A_4\xi^4 + \cdots \quad \text{(S1)}$$

where $\Phi_0(T)$, A_1, A_2, A_3, A_4, ... are the functions of T. Here, due to the symmetry, the expansion of Φ contains no odd-order terms, that is $A_1 = A_3 \equiv 0$. Then, the expansion of the thermodynamic has the form

$$\Phi(T,\xi) = \Phi_0(T) + A_2\xi^2 + A_4\xi^4 \quad \text{(S2)}$$

It is assumed that the function $A_2(T)$ has no singularity at the critical temperature, so that $A_2(T)$ could be expanded near the critical T_p

$$A_2(T) = b \times (T - T_p) \quad \text{(S3)}$$

The coefficient $A_4(T)$ may be replaced by $A_4(T_p)$. The expansion then becomes

$$\Phi(T,\xi) = \Phi_0(T) + b \times (T - T_p)\xi^2 + A_4\xi^4 \tag{S4}$$

The dependence of order parameter on temperature near the transition point is determined by equating the derivative $\partial\Phi/\partial\xi$ to zero

$$\xi^2 = -A_2/2A_4 = b \times (T_p - T)/2A_4 \tag{S5}$$

Neglecting the higher powers of ξ, the entropy in the unsymmetrical phase could be expressed as

$$S = -\frac{\partial\Phi}{\partial T} = S_0 + \frac{b^2}{2A_4}(T - T_p) \tag{S6}$$

Finally, the specific heats $C_p = T(\partial S/\partial T)_P$ for the unsymmetrical phase could be obtained by differentiating the equation

$$C_p = C_{p0} + \frac{b^2 T_p}{2A_4} \tag{S7}$$

For symmetrical phase, $S = S_0$. Therefore, $C_p = C_{p0}$.

B: Impact of phonon softening on electrical resistivity

Katayama et al.[3, 4] found that the enhanced resistivity near T_p was mainly caused by the scattering of carriers from soft transverse-optic (TO) phonons, while the carrier—acoustic phonon and carrier—defect interactions gave rise to simply a smooth background. The extra electrical resistivity $\Delta\rho_2$ caused by the scattering of carriers from soft TO phonons is closely related to the phonon frequency ω and given by

$$\Delta\rho_2 = \frac{3\pi\hbar k_B T}{32e^2\overline{M}D}\left|\frac{V_P}{a}\right|^2 E_F^{-2}\left[1 - \frac{\omega^2}{\gamma}ln(1 + \frac{\gamma}{\omega^2})\right] \tag{S8}$$

where $\overline{M}$ is the reduced mass density per unit cell, V_P is the potential energy of electron-phonon interaction, a is the lattice parameter, and γ is related to the

dispersion coefficient D and Fermi vector of electrons k_F, which is given by $\gamma = 4Dk_F^2$. The ratio of b to γ, i.e., b/γ characterizes the curvature in the resistivity caused by the soft-TO phonon.[5] As can be seen that Katayama did not establish a relationship between the order parameter and resistivity, nor did he uncover the impact of phonon softening on the Seebeck coefficient. Based on the standard theory of the second-order phase transition, the TO phonon frequency ω decreases rapidly when T is approaching to T_p (**Figure 1d**) [6]

$$\omega^2 = b(T_p - T) \tag{S9}$$

Under the relaxation time approximation, the electrical resistivity ρ_0 for a normal degenerate semiconductor is expressed as:

$$\rho_0 = \frac{3}{8\pi e^2 m^{*\frac{1}{2}}}\left(\frac{\hbar^2}{2}\right)^{\frac{3}{2}}\tau_0^{-1}E_F^{-s-\frac{3}{2}} \tag{S10}$$

Here, m^* is the carrier effective mass, τ_0 is the proportionality constant, s represents the scattering factor and takes the value of -0.5 for acoustic phonon scattering and 0.5 for optical phonon scattering. Combining equations (S8-10), we obtain the ratio of $\Delta\rho_2$ to ρ_0

$$\frac{\Delta\rho_2}{\rho_0} = C_1 T\left[1 - \frac{b(T_C - T)}{\gamma} ln\left(1 + \frac{\gamma}{b(T_C - T)}\right)\right] \tag{S11}$$

where $C_1 = \frac{\sqrt{2}}{16}\frac{k_B}{\bar{M}D}\left|\frac{V_p}{a}\right|^2 \frac{m^{*\frac{1}{2}}\tau_0 E_F^{-\frac{1}{2}+s}}{\hbar^2}$ is a parameter independent on temperature T and phase transition parameter b.

C: Impact of phonon softening on Seebeck coefficient

The ratio of $\Delta\rho_2$ to ρ_0 is given by

$$\frac{\Delta\rho_2}{\rho_0} = C_0 E_F^{-\frac{1}{2}+s} G' \tag{S12}$$

where $C_0 = \frac{\sqrt{2}}{16}\frac{k_B}{\overline{M}D}\left|\frac{v_{T_0}}{a}\right|^2 \frac{m^{*\frac{1}{2}}\tau_0}{\hbar^2} T$ is a parameter independent on the carrier energy, $G' = [1 - \frac{b(T_C - T)}{4Dk_F^2} ln(1 + \frac{4Dk_F^2}{b(T_C - T)})]$ is related to the temperature *T* and phase transition parameter *b*, as well as the Fermi wave vector k_F. As we know, k_F is related to the Fermi energy E_F through

$$E_F = \frac{\hbar^2 k_F^2}{2m_e}$$

Then G' becomes

$$G' = [1 - \frac{b(T_C - T)\hbar^2}{8Dm_e}\frac{1}{E_F} ln(1 + \frac{8Dm_e E_F}{b(T_C - T)\hbar^2})]$$

$$= [1 - \frac{C_1}{E_F} ln(1 + \frac{E_F}{C_1})] \tag{S13}$$

Here, $C_1 = \frac{b(T_C - T)\hbar^2}{8Dm_e}$, has an extremely low value, almost approaching zero.

According to the Mott expression, the total Seebeck coefficient α is

$$\alpha = \frac{\pi^2 \kappa_B^2 T}{3e}\left\{-\frac{d(ln\,\rho)}{dE}\right\} \tag{S14}$$

By substitution of eq. S12 into S14, we obtained

$$\alpha = \frac{\pi^2 \kappa_B^2 T}{3e}\left\{-\frac{d\left[\ln(\rho_0 + \rho_0 C_0 E_F^{-\frac{1}{2}+s} G')\right]}{dE}\right\}$$

$$= \frac{\pi^2 \kappa_B^2 T}{3e} \left\{ -\frac{d\ln\rho_0}{dE} - \frac{d\left[\ln(1 + C_0 E_F^{-\frac{1}{2}+s} G')\right]}{dE} \right\} \tag{S15}$$

$$= \alpha_0 + \Delta\alpha_2$$

Here, the first term $\alpha_0 = \frac{\pi^2 \kappa_B^2 T}{3e} \{-\frac{d\ln\rho_0}{dE}\}$ is the Seebeck coefficient for the normal phase, the second term $\Delta\alpha_2$ is the enhanced Seebeck coefficient contributed by the phonon softening.

$$\Delta\alpha_2 = \frac{\pi^2 \kappa_B^2 T}{3e} \left\{ -\frac{d\left[\ln(1 + C_0 E_F^{-\frac{1}{2}+s} G')\right]}{dE} \right\}$$

$$= \frac{\pi^2 \kappa_B^2 T}{3e} \frac{1}{1 + C_0 E_F^{-\frac{1}{2}+s} G'} \frac{-C_0 d(E_F^{-\frac{1}{2}+s} G')}{dE}$$

$$= \frac{-\pi^2 \kappa_B^2 T}{3e} \frac{C_0 E_F^{-\frac{1}{2}+s} G'}{1 + C_0 E_F^{-\frac{1}{2}+s} G'} \frac{d\left[\ln\left(E_F^{-\frac{1}{2}+s} G'\right)\right]}{dE}$$

$$= \frac{-\pi^2 \kappa_B^2 T}{3e} \frac{\Delta\rho_2}{\rho_0 + \Delta\rho_2} \frac{d\left[\ln\left(E_F^{-\frac{1}{2}+s} G'\right)\right]}{dE}$$

$$= \frac{-\pi^2 \kappa_B^2 T}{3e} \frac{\Delta\rho_2}{\rho_0 + \Delta\rho_2} \frac{1}{E_F} [-\frac{3}{2} + \lambda + \frac{E_F^2}{\left[E_F - C_1 \ln(1 + \frac{E_F}{C_1})\right](C_1 + E_F)}]$$

Since C_1 is almost approaching to zero, the term $C_1 \ln(1+\frac{E_F}{C_1})$ is also approaching to zero. Therefore, $\Delta\alpha_2$ is simplified to

$$\Delta\alpha_2 = \frac{\pi^2 \kappa_B^2 T}{3e} \frac{\Delta\rho_2}{\rho_0 + \Delta\rho_2} \frac{1}{E_F} (\frac{1}{2} - s)$$

Under the assumption of single parabolic band, the Seebeck coefficient α_0 for a normal degenerate semiconductor is

$$\alpha_0 = \frac{\pi^2}{3} \frac{{\kappa_B}^2 T}{e} \cdot \frac{s + \frac{3}{2}}{E_F}$$

Therefore, the ratio of $\Delta\alpha_2$ to α_0 is given by

$$\frac{\Delta\alpha_2}{\alpha_0} = \frac{\Delta\rho_2/\rho_0}{1 + \Delta\rho_2/\rho_0} \frac{\frac{1}{2} - s}{s + \frac{3}{2}} \quad \text{(S16)}$$

D: Calculation of thermal conductivity during phase transitions[7, 8]

According to Chen et al.[7, 8], the approximate solution of the true thermal diffusivity λ_0 is

$$\frac{\lambda_m}{\lambda_0} \approx \frac{1}{1 + C_{pt}/C_{p0}} + \frac{C_{pt}/C_{p0}}{1 + C_{pt}/C_{p0}} \exp(-\frac{1.81BL^2}{\lambda_0 \pi^2} \frac{1 + C_{pt}/C_{p0}}{1}) \quad \text{(S17)}$$

where C_{p0} is the heat capacity from phonons and electrons (e.g., approximately the Dulong–Petit value), C_{pt} is the extra heat capacity arising from phase transitions, B is the speed of phase transition, and L is the sample thickness. In this equation, λ_m/λ_0 is mainly related to C_{pt}/C_{p0} and B. The term C_{pt}/C_{p0} could be obtained from the measured heat capacity curves (**Supplementary Fig. 1b and Fig. 7**), while values of B could be calculated by fitting the heat flow curves measured at different heating rates (**Supplementary Fig. 8-9**). The fitting curves and fitting parameters are shown in **Supplementary Fig. 10**, **Table S2** and **Table S3**, respectively. Based on

the determined C_{pt}/C_{p0} and B, the true thermal diffusivity λ_0 during the phase transition is corrected. Besides, the heat capacity without the contribution of phase transition C_{p0} could be obtained from the baseline of measured heat capacity curves. Then the true heat conduction could be solved by

$$\kappa = C_{p0} \times d \times \lambda_0 \tag{S18}$$

The results are shown in **Fig. 4a**.

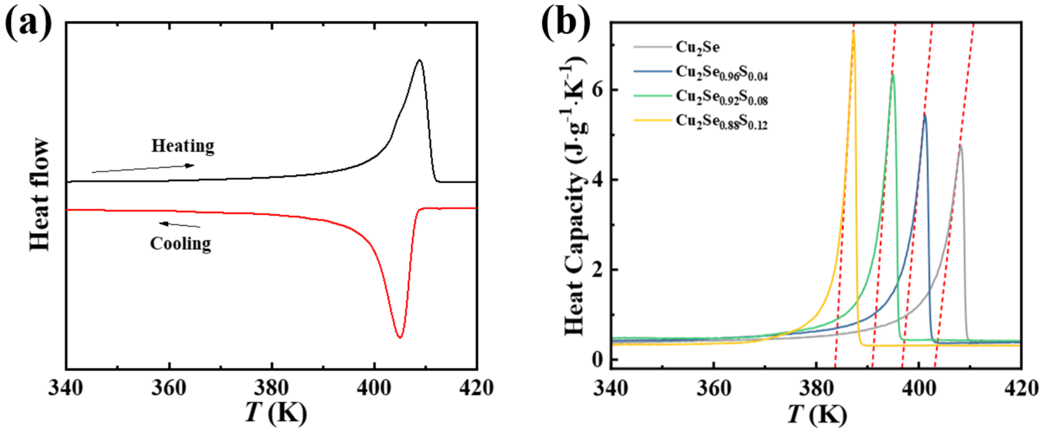

Fig. S1. Temperature dependence of heat flow and heat capacity. (a) Heat flow measured by the differential scanning calorimetric (DSC) method during heating and cooling for Cu_2Se. **(b)** Heat capacity for $Cu_2Se_{1-x}S_x$. The dash lines denote the tangents to C_p near the critical temperature.

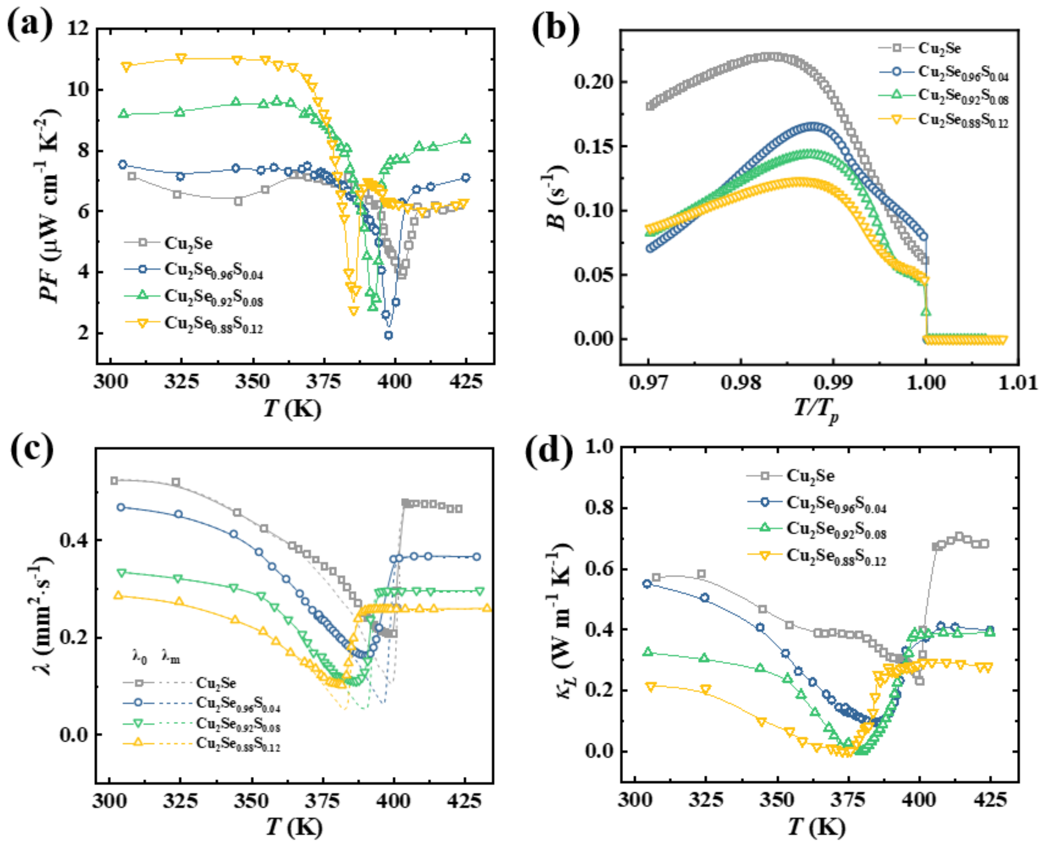

Fig. S2. Power factor *PF* and Thermal transport properties for $Cu_2Se_{1-x}S_x$. (a) Temperature dependence of power factor *PF* for $Cu_2Se_{1-x}S_x$ (x = 0, 0.04, 0.08 and 0.12). **(b)** The speed of phase transition *B* as a function of T/T_p. **(c)** Temperature dependence of measured thermal diffusivity λ_m and corrected thermal diffusivity λ_0. **(d)** Temperature dependence of lattice thermal conductivity κ_L.

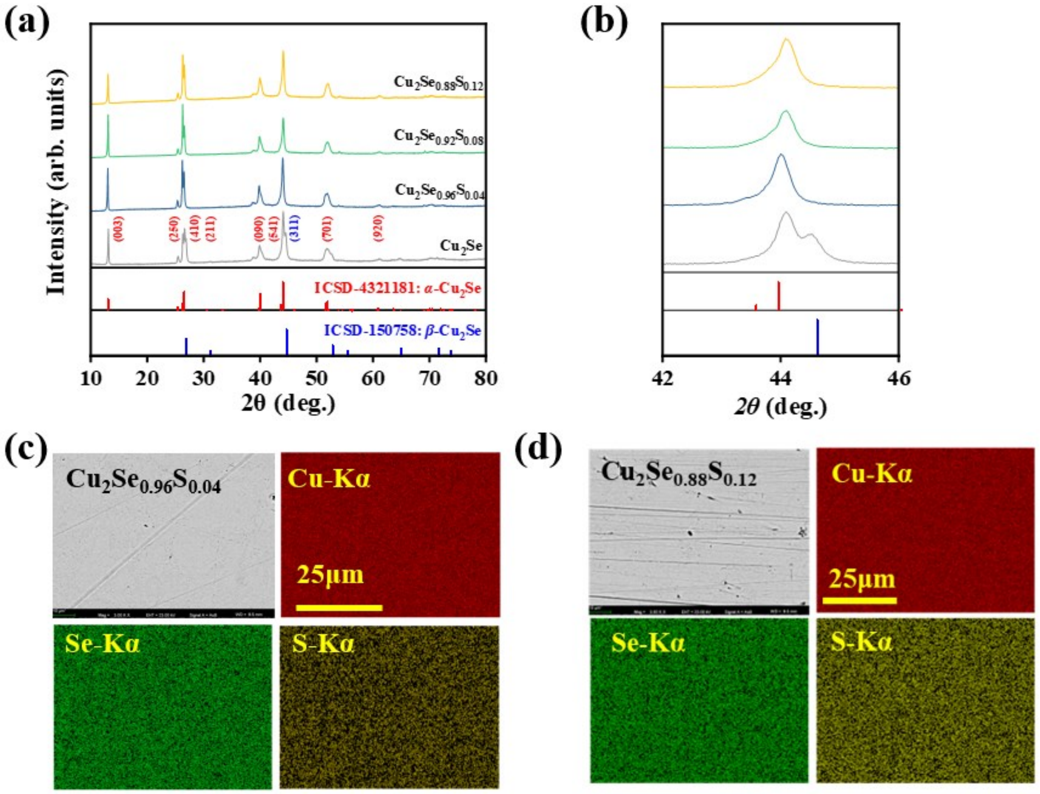

Fig. S3. X-ray diffraction (XRD) patterns and elemental energy dispersive spectroscopy (EDS) mapping for $Cu_2Se_{1-x}S_x$. (a) Room-temperature powder X-ray diffraction (XRD) patterns and **(b)** enlarged XRD patterns from $2\theta = 42°$ to 46°. Elemental energy dispersive spectroscopy (EDS) mapping for **(c)** $Cu_2Se_{0.96}S_{0.04}$ and **(d)** $Cu_2Se_{0.88}S_{0.12}$. Nearly all the diffraction peaks can be indexed to the Cu_2Se trigonal phase ($R\bar{3}m$). The weak diffraction peaks belonging to cubic phase ($Fm\bar{3}m$) in pristine Cu_2Se is ascribed to the severe copper precipitation during preparation. After alloying S in Cu_2Se, the copper precipitation is greatly suppressed due to the enhanced bonding energy between Cu and Se/S. As a result, single trigonal phase with homogenously distributed elements is obtained for $Cu_2Se_{1-x}S_x$ solid solutions.

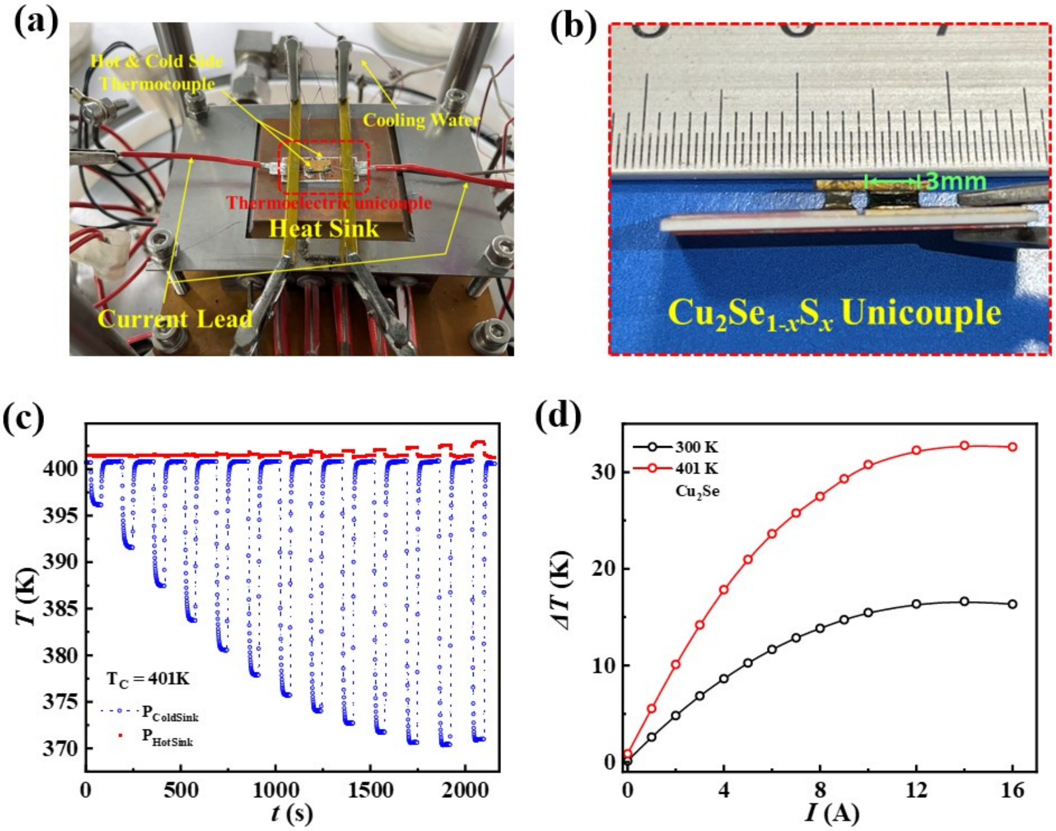

Fig. S4. Experimental setup and cooling ability of TE unicouple. (a) Experimental setup for the thermoelectric cooling measurement with **(b)** a unicouple consisting of p-type $Cu_2Se_{1-x}S_x$ and n-type $Yb_{0.3}Co_4Sb_{12}$. $Yb_{0.3}Co_4Sb_{12}$ is chosen as the n-type leg because its zT value is comparable with those of the normal trigonal and cubic phases of $Cu_2Se_{1-x}S_x$. **(c)** Temperature difference at different input current measured at T_C = 401K for $Cu_2Se/Yb_{0.3}Co_4Sb_{12}$ thermoelectric unicouple. The red squares represent the hot side temperature at the bottom of the p-leg while the blue circles represent the cold side temperature on the top of the p-leg. **(d)** Temperature drops as a function of input current. The maximum temperature drop measured at 401 K is 32.8 K.

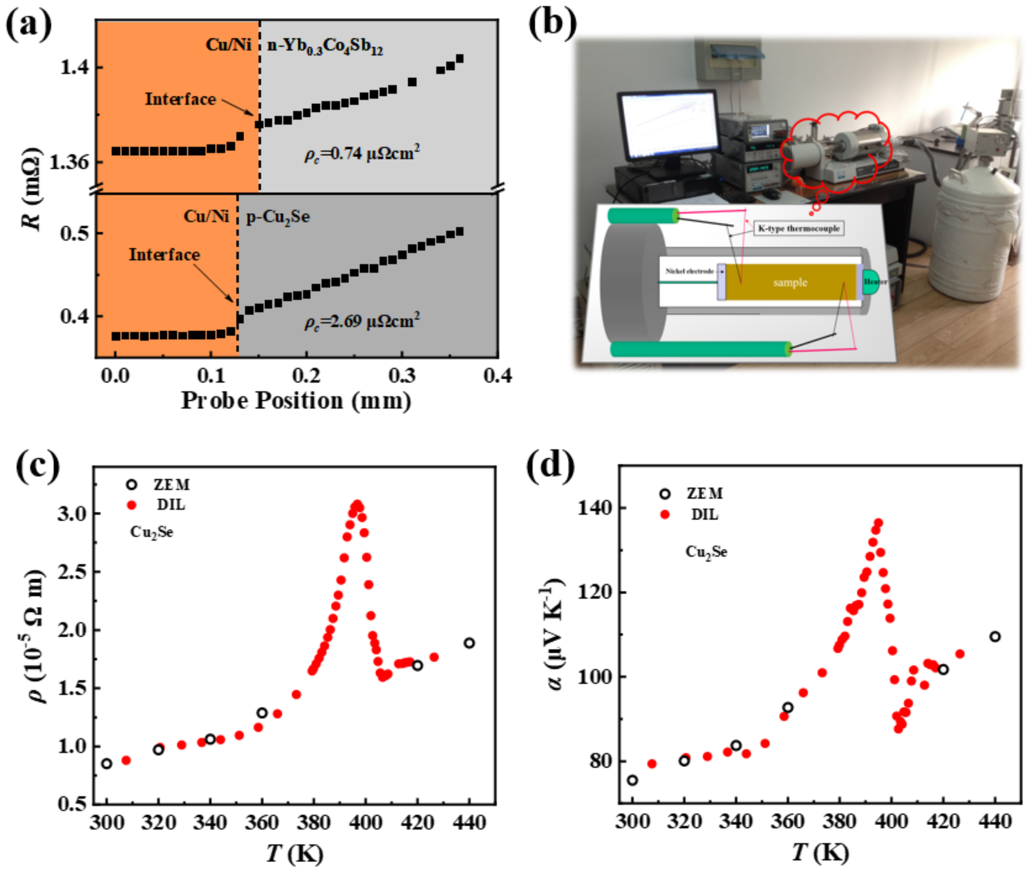

Fig. S5. **Contact resistivities and home-made equipment. (a)** Contact resistivities of n- and p-legs. **(b)** Schematic of the home-made equipment for measuring the Seebeck coefficient and electrical resistivity during the phase transition. Comparison of the **(c)** Electrical resistivity *ρ* and **(d)** Seebeck coefficient *α* measured by modified DIL (Red circle) and ULVAC ZEM-3 system (Black circle) for Cu_2Se.

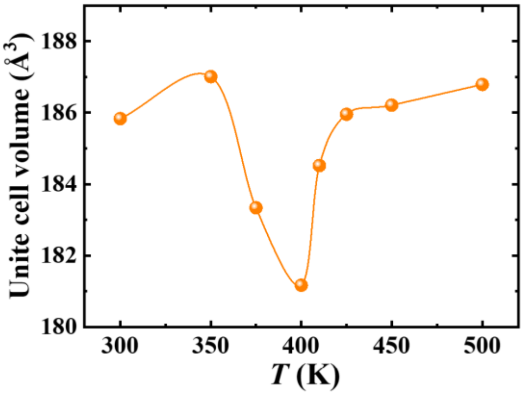

Fig. S6. The unit cell volume as a function of temperature.

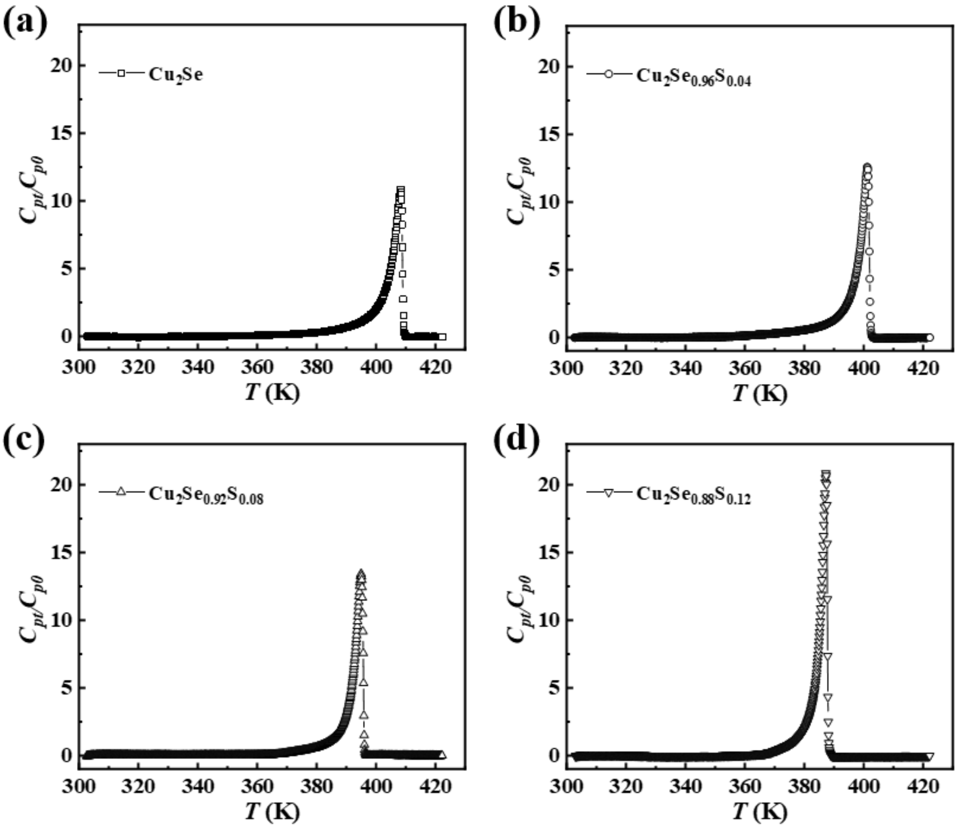

Fig. S7. The ratio of C_{pt} to C_{p0}. C_{pt}/C_{p0} measured for **(a)** Cu_2Se, **(b)** $Cu_2Se_{0.96}S_{0.04}$, **(c)** $Cu_2Se_{0.92}S_{0.08}$, and **(d)** $Cu_2Se_{0.88}S_{0.12}$ during the phase transition. The heat capacity is measured with a heating rate of 5 K min^{-1}.

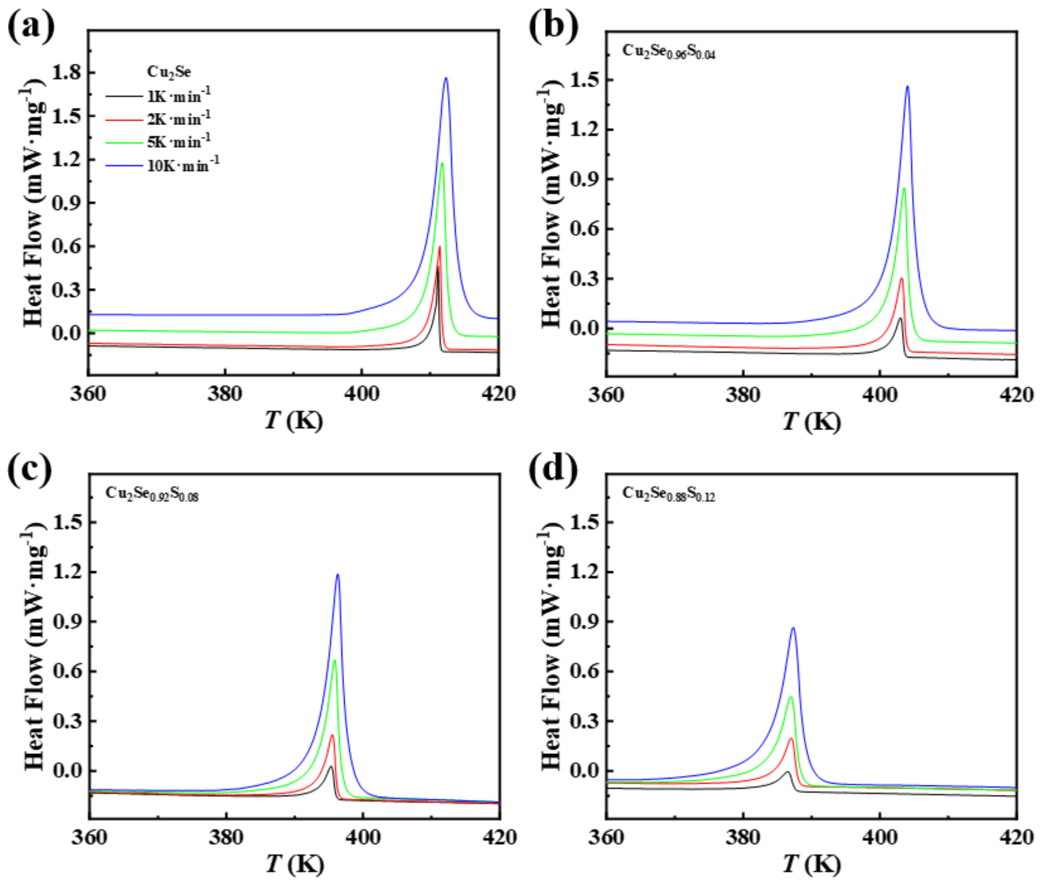

Fig. S8. **Heat flow curves measured by DSC with different heating rates (1.0, 2.0, 5.0 and 10 K min^{-1}).** Heat flow curves for **(a)** Cu_2Se, **(b)** $Cu_2Se_{0.96}S_{0.04}$, **(c)** $Cu_2Se_{0.92}S_{0.08}$, and **(d)** $Cu_2Se_{0.88}S_{0.12}$.

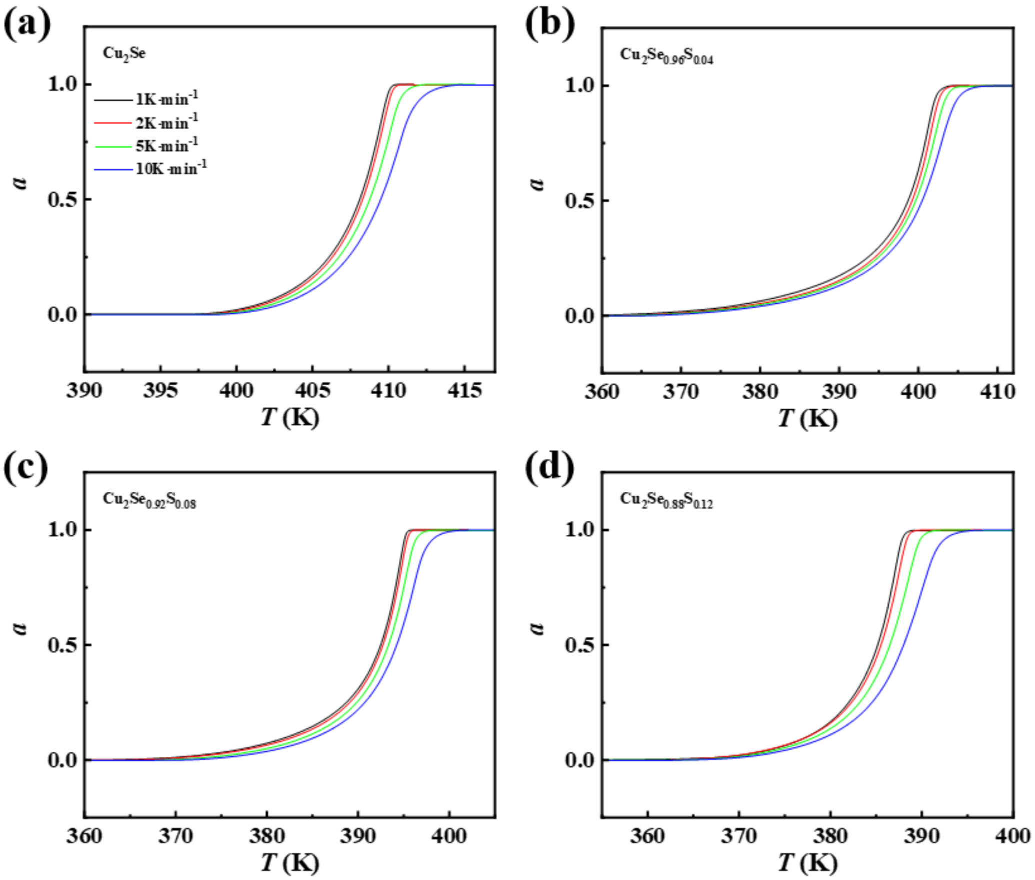

Fig. S9. **Temperature dependence of mole fraction of high temperature *β*-phase at different heating rates (1.0, 2.0, 5.0 and 10 K min^{-1}) for (a) Cu_2Se, (b) $Cu_2Se_{0.96}S_{0.04}$, (c) $Cu_2Se_{0.92}S_{0.08}$, and (d) $Cu_2Se_{0.88}S_{0.12}$.**

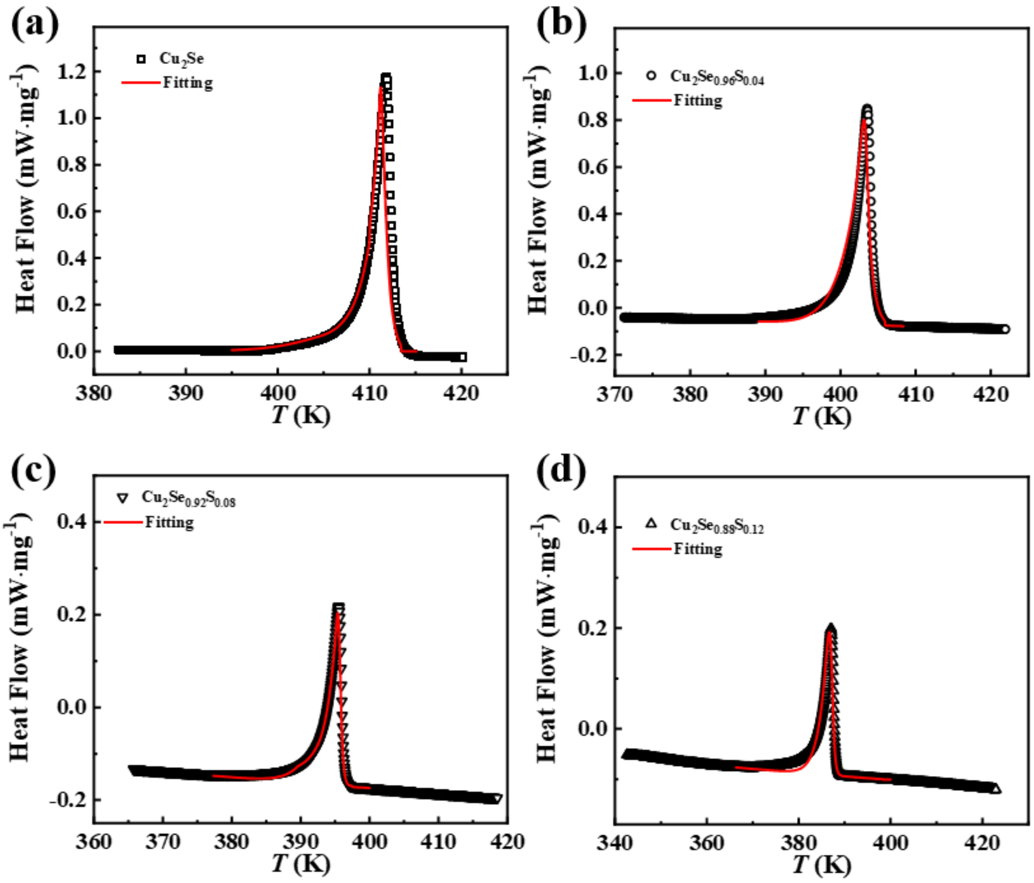

Fig. S10. **Heat flow measured by DSC and the fitted lines for (a) Cu_2Se, (b) $Cu_2Se_{0.96}S_{0.04}$, (c) $Cu_2Se_{0.92}S_{0.08}$, and (d) $Cu_2Se_{0.88}S_{0.12}$.**

Table S1. Fitted parameters by equation (7) and (9). C_1 is the parameter independent on temperature T and phase transition parameter b; b is the phase transition parameter; γ is related to the dispersion coefficient D and Fermi vector of electrons k_F, which is given by $\gamma = 4Dk_F^2$; c is the first derivate of the energy with respect to order parameter, s represents the scattering factor.

Materials	C_1 [K^{-1}]	b [10^{26}]	γ [10^{26} s^{-2}]	c [10^{14} K]	s
Cu_2Se	0.110	1.50	0.30	10.8	0.15
$Cu_2Se_{0.96}S_{0.04}$	0.108	1.66	0.45	5.0	0.15
$Cu_2Se_{0.92}S_{0.08}$	0.065	1.80	0.59	3.8	0.15
$Cu_2Se_{0.880}S_{0.12}$	0.045	1.95	0.60	3.1	0.15

Table S2. Fitted parameters for thermal conductivity. n is the reaction order, A is the pre-exponential factor, E is the activation energy and ΔH is the phase transition enthalpy.

Materials	E [kJ mol^{-1}]	*Log(A)*	*n-1*	ΔH [J g^{-1}]
Cu_2Se	287	35	0.08	32.83
$Cu_2Se_{0.96}S_{0.04}$	308	39	0.13	32.1
$Cu_2Se_{0.92}S_{0.08}$	324	41	0.16	31.3
$Cu_2Se_{0.880}S_{0.12}$	352	46	0.18	30.73

Table S3. Measured critical temperature T_p using a heating rate of 1 K min^{-1} and the measured increment of critical temperature ΔT_p using the heating rate of 2 K min^{-1}, 5 K min^{-1}, and 10 K min^{-1} for $Cu_2Se_{1-x}S_x$ (x = 0, 0.04, 0.08 and 0.12).

Materials	T_p (K)	ΔT_p (K)		
	1K min^{-1}	2K min^{-1}	5K min^{-1}	10K min^{-1}
Cu_2Se	409.5	0.2	0.6	1.2
$Cu_2Se_{0.96}S_{0.04}$	401.3	0.2	0.9	1.7
$Cu_2Se_{0.92}S_{0.08}$	394.6	0.2	0.7	1.7
$Cu_2Se_{0.880}S_{0.12}$	387.1	0.5	1.6	3

References

1. L. D. Landau, E. M. Lifshitz and L. E. Reichl, *Phys Today*, 1981, **34**, 74-74.
2. M. Fujimoto, *The Physics of Structural Phase Transitions*, The Physics of Structural Phase Transitions, 2005.
3. S. Katayama, *Solid State Commun.*, 1976, **19**, 381-383.
4. S. Katayama and D. Mills, *Phys Rev B*, 1980, **22**, 336.
5. I. Avramova and S. Plachkova, *J. Phys. Condens. Matter* 2001, **13**, 43.
6. C. N. R. Rao, 1978.
7. H. Chen, Z. Yue, D. Ren, H. Zeng, T. Wei, K. Zhao, R. Yang, P. Qiu, L. Chen and X. Shi, *Adv. Mater.*, 2018, **31**.
8. A. E, *Annual book of ASTM standards*, 1991, **15**, 750-757.
